# Community screening for hepatitis C virus infection in a low-prevalence population

**DOI:** 10.1186/s12889-019-7388-7

**Published:** 2019-08-02

**Authors:** Karen K. Kyuregyan, Elena Yu. Malinnikova, Natalia V. Soboleva, Olga V. Isaeva, Anastasia A. Karlsen, Vera S. Kichatova, Ilya A. Potemkin, Elena V. Schibrik, Olga A. Gadjieva, Boris A. Bashiryan, Natalya N. Lebedeva, Igor L. Serkov, Anna Yankina, Claudio Galli, Mikhail I. Mikhailov

**Affiliations:** 1 Department of Viral Hepatitis, Russian Medical Academy of Continuing Professional Education, 125993 Moscow, Russia; 2 Mechnikov Research Institute for Vaccines and Sera, 105064 Moscow, Russia; 3 Belgorod regional center for disease control and prevention, 308023 Belgorod, Russia; 4 BurdenkoNational Medical Research Center of Neurosurgery, 125047 Moscow, Russia; 5 Moscow Regional Center for the Prevention and Control of AIDS and Infectious Diseases, 129110 Moscow, Russia; 6 Medical Communications, Abbott Diagnostics, 125171 Moscow, Russia; 7 Medical Affairs Infectious Diseases, Abbott Diagnostics, 00144 Rome, Italy

**Keywords:** Hepatitis C, Screening, Age cohorts, HCV antigen test

## Abstract

**Background:**

Age cohort screening for hepatitis C virus (HCV) might be an effective strategy if the majority of undiagnosed cases are concentrated in a particular age group. The objective of this study was to determine HCV prevalence in different age cohorts of the general population in the Central European part of Russia and second, to assess feasibility of HCV antigen testing for community screening programs.

**Methods:**

Sera from 2027 volunteers were tested for anti-HCV (Architect Anti-HCV, Abbott Laboratories). All anti-HCV reactive samples were confirmed in an immunoblot and tested for HCV Ag (ARCHITECT HCV Ag, Abbott Laboratories), HCV RNA and HCV viral load.

**Results:**

Out of 31 individuals with anti-HCV reactive result, 22 (71%) were confirmed by immunoblot, six were false positives and three were indeterminate. Active infection was observed in 73% of anti-HCV confirmed positives. Five out of 16 individuals had low HCV-RNA levels (< 10,000 IU/mL) and one of those had a very low level (594 IU/mL). Agreement between HCV Ag and HCV RNA was 100%. Total anti-HCV and active HCV infection rates were 1.09% (22/2027) and 0.79% (16/2027), respectively. The peak rates were observed in people 60 years or older (anti-HCV: 2.84% [95% CI: 1.66–4.74%], 13/319; HCV RNA/HCV Ag: 2.23% [95% CI: 1.20–4.00%], 10/319).

**Conclusions:**

Overall HCV prevalence is low, except in people 60 years or older. The latter should be considered as a target group for HCV screening. The high agreement between HCV RNA and HCV Ag suggests the utility of HCV Ag testing to confirm active infection in screening programs.

## Background

Current antiviral regimens can cure up to 95% of persons with HCV infection and thus reduce the risk of death from cancer and liver cirrhosis, as well as the number of new infections [1]. This means that screening and timely treatment of chronic hepatitis C (CHC) are the most promising measures in combating the epidemic of this infection [2]. Eastern Europe and Central Asia (EECA) are among the regions with the highest prevalence of HCV infection [3]. Within the EECA, Russia has the highest absolute number of infections and, together with Egypt, China, India, Nigeria and Pakistan, accounts for more than half of the global HCV burden [4]. According to the Russian State Statistical Observation service, the incidence of CHC in Russia has ranged between 32 and 40 cases per 100,000 population over the last 10 years [5]. However, the reported incidence rates do not fully reflect the HCV burden on the population. HCV infection can be asymptomatic for decades; as a result up to 90% of HCV-infected people are not currently identified and do not know their status [6]. In Russia, 3 to 5 million people are estimated to be infected with HCV [7]. Importantly, data on true HCV prevalence in Russia are lacking, as current estimates are based mostly on studies conducted in megalopolises such as Moscow and St. Petersburg, regions outside the Central European part of the country [8–10] or in specific cohorts (first time blood donors) and risk groups [5, 11]. To assess the true prevalence of HCV infection and to understand the epidemiological features of the infection in different territories, population-based studies are necessary. Depending on the characteristics of epidemiology in a particular region, HCV infection may be concentrated in certain age groups [12]. In this case, birth cohort screening is recommended as a useful tool to combat hidden HCV epidemics [13].

The screening marker of HCV infection is antibodies to the hepatitis C virus (anti-HCV) [14]. A significant proportion (up to 40–45%) of individuals with anti-HCV does not have current HCV infection and, thus, does not need antiviral therapy [15]. Molecular methods of detecting viral RNA are used to confirm active HCV infection [14]. An alternative method for confirming active HCV infection is the detection of a viral antigen in immunological tests [16]. Current recommendations for HCV screening in Russia suggest testing for anti-HCV in the following categories: pregnant women; blood donors; health workers; hemodialysis patients; patients undergoing surgery or chemotherapy (before hospitalization); people living with HIV; patients with tuberculosis; individuals from risk groups (PWID, men who have sex with men (MSM), sex workers); and prison inmates (upon incarceration). These recommendations are important for prevention of HCV infection, but, are not comprehensive enough to fully identify all infected people. WHO recommends performing population-based serosurveys to assess the HCV burden in high-risk groups and in the general population in order to develop screening, care and treatment strategies [17, 18]. Russia has one of the highest HCV positivity rates in risk groups: up to 71% in PWID [11]. But, due to limited harm reduction programs for PWID in Russia and the criminalization of drug use, this major risk group often avoids HCV screening programs. On the other hand, the global HCV epidemic has gone beyond risk groups. Thus, epidemiological surveys in the general population are urgently needed. The Central European Region of Russia (CER) accounts for more than 25% of the country’s inhabitants with the highest population density, according to the Bulletin of the Russian Statistics Agency [http://www.gks.ru/free_doc/new_site/population/demo/Popul2019.xls]. With the exception of Moscow, the population in this region lives mainly in small cities with no more than 100,000 inhabitants. Belgorod Region, located in the southwest of CER, is typical of the whole area in terms of economic and demographic characteristics. Registered incidence of CHC in CER is consistently lower than in the northwestern and Asian parts of the country, and lower than the Russian average. CHC incidence in Belgorod Region is also 20–25% lower compared to the average Russian indicators. Thus, Belgorod Region, with its population ca. 1.5 million, is a fairly representative region in terms of estimating the prevalence of HCV in the general population in CER. The objective of this study was to determine HCV prevalence in different age cohorts of the general population in the Belgorod Region of the Russian Federation and second, to assess the feasibility of HCV antigen testing for community screening programs.

## Methods

### Study design and population

The prospective seroprevalence study was conducted in Belgorod region of the Russian Federation between March to September 2018. In total 2027 people were surveyed, which is about 0.13% of the population of Belgorod Region (1,547,418 people in 2018 according to the Bulletin of the Russian Statistics Agency [http://www.gks.ru/free_doc/new_site/population/demo/Popul2019.xls]). Each participant completed a questionnaire with demographic data and risk factors for HCV infection. The study was conducted according to the principles expressed in the Declaration of Helsinki. Written informed consent was obtained from all participants. The study design was approved by the Ethics Committee of the Mechnikov Research Institute for Vaccines and Sera, Moscow, Russia (Approval #1 dated 2018-28-02).

The volunteers were persons undergoing routine medical examinations, vaccine office visitors undergoing routine vaccinations, and patients visiting the polyclinic for reasons not related to infectious diseases. Inclusion criteria were a signed and dated informed consent form (ICF) approved by the Ethics Committee. Subjects were males or females 0–95 years of age, apparently healthy people with no symptoms of acute disease at enrollment (self-reported or parent-reported) and permanent residents in the study region. Exclusion criteria were treatment with blood products within 3 months before entering the study (self-reported or parent-reported), and a body temperature over 37.10 °C or acute illnesses.

The study included eight age groups (ages 1–14, 15–19, 20–29, 30–39, 40–49, 50–59, 60–69 and ≥ 70 years). The population sample size was calculated for the known size of the general population of Belgorod region based on the estimated anti-HCV prevalence rates in the Russian Federation to be about 4% according to published data [5] with chosen power (80%) and confidence level (95%) [19]. Sample size in all age groups was chosen to be the same to determine HCV prevalence in each group with the same level of accuracy. The mean population sample size in each age group was 250 individuals (174–319).

Venous blood samples from ulnar vein were collected using BD Vacutainer® Plus tubes (Becton Dickinson, USA) by trained nurse in equipped and licensed procedure room for blood collection using standard procedure. Blood samples 8 mL in volume were obtained from adults and adolescents, while from children under 15 years we obtained 3 mL. All sera samples were coded and aliquoted, and aliquots were stored at − 70 °C until testing.

### HCV testing

All serum samples were tested for anti-HCV by the Architect Anti-HCV test (Abbott Laboratories). The presence of anti-HCV was expressed as a signal-to-cutoff ratio (S/CO). Values > 1.0 were interpreted as reactive and between 0.80 and 0.99 as grey zone (GZ). All samples reactive or GZ in the screening test were confirmed by an immunoblot for antibodies to structural and non-structural HCV proteins (INNO-LIA HCV, Fujirebio Europe N.V.). Furthermore, all anti-HCV reactive and GZ samples were tested in parallel for HCV Ag in the ARCHITECT HCV Ag test (Abbott Laboratories) and for HCV RNA by two assays: AmpliSens®HCV-FL (Interlabservice, Russia) for the qualitative determination of HCV RNA with a sensitivity of 10 IU/mL, and Abbott RealTime HCV (Abbott Laboratories) with a sensitivity of 12 IU/mL for the determination of viral load. All testing procedures and interpretation of results were performed according to manufacturer’s instructions for the corresponding reagent kits.

In all samples positive for HCV RNA viral genotype was determined by amplifying and sequencing the core region of the HCV genome, as described elsewhere [20].

### Statistical analysis

Data analysis was performed using graphpad.com. Statistical analysis includes assessing the significance of differences of mean values between groups using Fisher’s exact test and chi-squared distribution with Yates correction (significance threshold *p* < 0.05). Linear regression analysis was used to assess the linear association between the HCV Ag and HCV RNA concentrations.

## Results

Total 2027 healthy volunteers participated in the prospective seroprevalence study in Belgorod region. Demographically, 930 (45.9%) of the volunteers were males and 1097 (54.1%) were females. The male/female ratio varied from 1:0.8 to 1:1.5 depending on age cohort. The mean age of participants in the whole cohort of volunteers was 42.2 years (SD = 24.6 years). The rural/urban population ratio was 1:6.5.

Out of 2027 serum samples, 27 were reactive in the screening test for anti-HCV (1.33%) and 4 were GZ (0.20%). Out of these 31 samples, 22 (71%) were confirmed by Inno-LIA, 6 were not confirmed (false positive) and 3 were indeterminate. Excluding the latter, the specificity of the anti-HCV assay was 99.70%, which is in accordance with the assay specifications.

The results of the qualitative and quantitative determination of HCV RNA, HCV Ag and HCV genotype in samples reactive or GZ for anti-HCV test are summarized in Table 1.

**Table 1:** Results of HCV RNA and HCV Ag testing in anti-HCV reactive serum samples

No.	Sample ID	Age group, years	Sex of participant	Anti-HCV S/CO	Inno-LIA result	HCV Ag, fmol/L	HCV RNA	HCV viral load, IU/mL	HCV genotype
1	94	50–59	F	13.10	Pos	3391.6	Pos	820,723	1b
2	99	40–49	F	2.88	Neg	0.01	Neg	Neg	n.t.
3	335	> 70	M	12.71	Pos	4.33	Pos	594	1b
4	386	60–69	F	1.46	Ind	0	Neg	Neg	n.t.
5	409	60–69	F	1.66	Ind	0	Neg	Neg	n.t.
6	472	> 70	F	15.20	Pos	0	Neg	Neg	n.t.
7	496	> 70	M	13.66	Pos	12,121.7	Pos	2,905,138	1b
8	506	> 70	M	1.41	Ind	0	Neg	Neg	n.t.
9	544	> 70	F	1.88	Pos	0.29	Neg	Neg	n.t.
10	578	> 70	F	11.08	Pos	51.23	Pos	7819	1b
11	582	> 70	F	13.10	Pos	621.62	Pos	167,552	1b
12	704	1–14	F	0.98	Neg	0	Neg	Neg	n.t.
13	908	> 70	M	4.30	Pos	0	Neg	Neg	n.t.
14	976	60–69	M	11.60	Pos	6013.27	Pos	743,465	1b
15	983	> 70	M	10.94	Pos	502.88	Pos	83,278	1b
16	1002	50–59	F	1.59	Pos	0	Neg	Neg	n.t.
17	1074	30–39	M	12.28	Pos	7.46	Pos	17,612	3a
18	1145	40–49	M	14.54	Pos	0	Neg	Neg	n.t.
19	1444^a^	20–29	F	1.01	Neg	(22.82)	Neg	Neg	n.t.
20	1517	50–59	M	14.29	Pos	0	Neg	Neg	n.t.
21	1529	50–59	M	11.51	Pos	8.92	Pos	3914	1b
22	1531	> 70	M	13.17	Pos	1906.12	Pos	385,515	1b
23	1541	50–59	M	12.14	Pos	7.54	Pos	4112	1b
24	1635	30–39	F	0.87	Neg	0	Neg	Neg	n.t.
25	1716	> 70	F	0.84	Neg	0	Neg	Neg	n.t.
26	1723	> 70	F	14.38	Pos	1496.22	Pos	320,851	1b
27	1729	> 70	F	12.04	Pos	5.08	Pos	2008	1b
28	1924	50–59	F	0.81	Neg	0	Neg	Neg	n.t.
29	2101	20–29	M	8.09	Pos	7201.39	Pos	995,879	3a
30	2018	> 70	F	13.21	Pos	2346.87	Pos	92,905	1b
31	2067	> 70	F	13.41	Pos	10,712.9	Pos	1,184,879	1b

*n.t.* not tested

^a^repeat testing for HCV Ag: 0.00 fml/L

None of the serum samples with a negative or indeterminate result in immunoblot was positive for HCV RNA or HCV Ag, except for one sample, ID 1444, which was negative at immunoblot and by both tests for HCV RNA, but gave a positive result for HCV Ag. However, re-testing for HCV Ag using another aliquot of sample ID 1444 yielded a negative result.

**Fig. 1:**
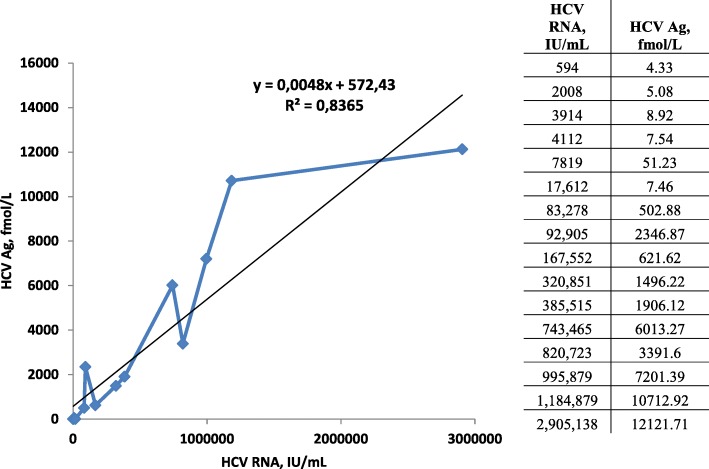
Correlation between HCV Ag and HCV RNA concentrations (*n* = 16)

HCV viral load ranged from 594 IU/mL to 2,905,138 IU/mL. Five out of 16 individuals had low HCV RNA levels (< 10,000 IU/mL) and one of those a very low level (594 IU/mL). HCV Ag was detected in all samples positive for HCV RNA, including samples with a low viral load. Thus, agreement between HCV Ag and HCV RNA was 100% when testing serum samples that were positive for anti-HCV. HCV Ag had good correlation with HCV-RNA [R^2^ = 0.84, *P* < 0.005] (Fig. 1).

Data on positivity rates for anti-HCV and active HCV infection, determined as positivity for HCV RNA and/or HCV Ag, are shown in Table 2.

**Table 2:** Frequency rates of anti-HCV and active HCV infection in general population of Belgorod Region (Russia)

Age group, years	N tested	Anti-HCV positive, N (%)	Active HCV infection, N (%)	% of active infection among anti-HCV positives
1–14	250	0 (0.00%)	0 (0.00%)	n.a.
15–19	266	0 (0.00%)	0 (0.00%)	n.a.
20–29	258	1 (0.39%)	1 (0.39%)	100.0%
30–39	260	1 (0.38%)	1 (0.38%)	100.0%
40–49	261	1 (0.38%)	0 (0.00%)	0.0%
50–59	239	5 (2.09%)	3 (1.26%)	60.0%
60–69	174	1 (0.57%)	1 (0.57%)	100.0%
> 70	319	13 (4.08%)	10 (3.13%)	76.9%
Total	2027	22 (1.08%)	16 (0.79%)	72.7%

On average, the detection rate of anti-HCV in the surveyed cohort was 1.09% (22/2027). In children aged 1–14 years and adolescents aged 15–19 years no anti-HCV positive cases were detected. The highest prevalence rates of anti-HCV were observed among persons over 70 years (4.08%, 13/319) and in the 50–59 age group (2.09%, 5/239), which significantly exceeded those observed in other age groups (*p* < 0.05). The distribution of men and women among anti-HCV-positive individuals was similar (12 men, 10 women). The prevalence of active HCV infection was 0.79% (16/2027), and the proportion of people with active infection among anti-HCV positives was 72.7% (16/22). Genotype 1b was absolutely prevalent (14/16, or 87.5%). The distribution of men and women among people with active HCV infection was approximately the same (9 men, 7 women). Cases of active HCV infection were not observed in the age groups 1–14, 15–19, and 40–49 years. The highest prevalence of active HCV infection was observed in the over-70 age group: 3.13% (10/319), which significantly exceeded corresponding figures among people younger than 50 (*p* < 0.05). Due to very low positivity rates in younger participants, which led to large confidence intervals for prevalence, all participants were grouped into three age cohorts: < 30, 30–59, and ≥ 60 years. HCV prevalence data in these cohorts are summarized in Table 3.

**Table 3:** Age-specific HCV prevalence rates in general population of Belgorod Region (Russia)

Age group, years	N tested	Anti-HCV positive		Active HCV infection		% of active infection among anti-HCV positives
		N	(%)	N	(%)	
< 30	774	1	0.13% [95% CI: 0.01–0.81%]	1	0.13% [95% CI:0.01–0.81%]	100.0%
30–59	760	7	0.92% [95% CI:0.41–1.93%]	4	0.53% [95% CI:0.15%-1,40%]	57.1%
≥60	493	14	2.84% [95% CI:1.66–4.74%]	11	2.23% [95% CI:1.20–4.00%]	78.6%

The frequency of HCV seropositivity and active HCV infection both peaked in the ≥60 years age group (*p* < 0.05 when compared to age groups < 30 and 30–59 years), and was almost absent in people aged under 30.

**Fig. 2:**
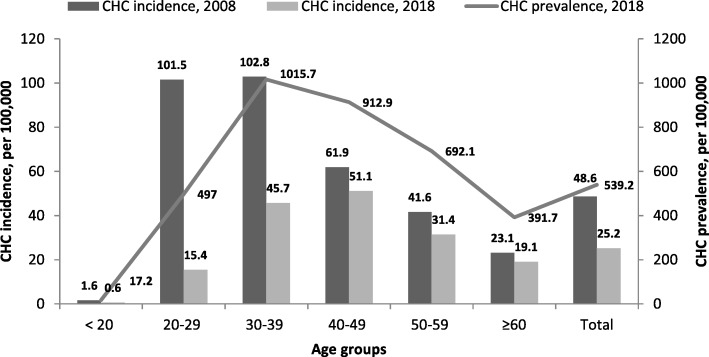
Age-specific rates of registered CHC prevalence and incidence in Belgorod Region (Russia)

To understand the age-specific distribution of diagnosed cases of HCV infection in the region studied, we analyzed the incidence and morbidity data from Belgorod Regional Center for Disease Control and Prevention. Age-specific CHC prevalence in Belgorod Region (the total number of cases identified from the beginning of registration in 1999 until the end of 2018) as well as incidence rates registered in 2008 and 2018, both per 100,000, are shown in Fig. 2.

The peak CHC incidence shifted over 10 years from the 20–29 and 30–39 age groups in 2008 to 30–39 and 40–49 in 2018. Both incidence and prevalence rates were highest in the 30–39 age group and low in those over 60 years.

## Discussion

The prevalence data obtained in our study were surprisingly low. The overall HCV prevalence in Russia has been estimated to be as high as 3.3% [21], which is about four times higher than data from this study. In our survey, rates of HCV infection are disproportionately higher in older individuals, born after 1958 (age over 60). The same is frequently observed in many regions of the world [22–24]. Contrary to the observed HCV prevalence pattern in the general population, the registered incidence of CHC in Belgorod Region peaked in younger people (ages 30–39) during last 10 years. Combined with HCV prevalence rates from our serosurvey, the incidence data suggest that a large proportion of HCV-infected people younger than 60 are already identified using existing screening programs, but older people definitely are not sufficiently covered by diagnostics. The high rates of registered CHC incidence in younger adults might be the explanation for low positivity rates in the general population overall and in younger age groups observed in our survey, indicating a ‘depletion’ of unidentified HCV cases in people younger than 60.

Interestingly, a study conducted recently in Yakutia, a vast but sparsely populated region in the Asian part of Russia, demonstrated a drop in anti-HCV prevalence in the general population, from 4.0 to 2.0% over 10 years (2008–2018). The observed drop in seroprevalence was associated with a decrease in seropositivity rates in the 30–39 age group (9.6% in 2008 vs. 1.7% in 2018, *p* < 0.05), while anti-HCV rates in people aged 60 years or older remained the same (12.1% in 2008 and 9.9% in 2018, *p* > 0.05) [25]. Perhaps the results of that study cannot be compared directly with data from this current study, as different diagnostic kits were used for detection of HCV markers, but the overall trend is clear.

The search for cheaper and faster diagnostic algorithms for confirmation of active HCV infection for screening programs is an important task. The WHO and the European Association for the Study of the Liver (EASL) have stated that chronic HCV infection can be diagnosed by either HCV RNA or HCV Ag positivity [14, 26]. The high overall agreement between HCV RNA and HCV Ag observed in a number of studies suggests that the latter could be used as an alternative to HCV RNA testing in population screening [27–29]. This approach is especially useful for reflex testing performed on the same platform and in the same sample as for the initial antibody screening test. A limitation of this strategy may be lower sensitivity of HCV Ag compared to HCV RNA testing, but in our study both approaches gave 100% agreement, even though about one third of samples had a viral load less than 10^5^ IU/mL, and one of those had very low RNA concentration (< 10^3^ IU/mL). HCV screening in elderly people might be complicated by an unexpectedly high proportion of patients with low viral load. The results of a cross-sectional study of elderly HCV outpatients demonstrated that viral load was significantly lower in those aged 75 and over compared to the “early elderly” group aged 65–74 [30]. It should be noted that in our study three out of five patients with low viral load were over 80. Nevertheless, our data demonstrated substantial equivalence between HCV RNA and HCV Ag for screening the population where the majority of infection cases are concentrated in the elderly.

The limitations of this study are related to the low rate of HCV positivity, which leads to quite large confidence intervals for prevalence estimates. Furthermore, some active infections may have been missed in anti-HCV negative samples (early phase, immunocompromised).

## Conclusions

While HCV prevalence in Russia remains to be fully elucidated, the results obtained in the Central European part of country suggest that the overall prevalence is low. The frequency of HCV positive cases increases with age, being most prevalent in people aged 60 or older. The latter constitute a risk group for HCV infection and should be considered as a target group for HCV screening. The high agreement between HCV RNA and HCV Ag suggests the utility of HCV Ag testing to confirm active infection in screening programs.

## Data Availability

The datasets used and/or analyzed during the current study are available from the corresponding author on reasonable request.

## Abbreviations

CER: Central European Region of Russia
CHC: Chronic hepatitis C
EASL: Study of the Liver
EECA: Eastern Europe and Central Asia
GZ: Grey zone
HCV Ag: Hepatitis C virus antigen
HCV: Hepatitis C virus
MSM: Men who have sex with men
PWID: People who inject drugs
STD: Sexually transmitted disease
WHO: World Health Organization

## References

[1] Falade-Nwulia O, Suarez-Cuervo C, Nelson DR, Fried MW, Segal JB, Sulkowski MS (2017). Oral direct-acting agent therapy for hepatitis C virus infection: a systematic review. Ann Intern Med.

[2] Durham DP, Skrip LA, Bruce RD, Vilarinho S, Elbasha EH, Galvani AP, Townsend JP (2016). The impact of enhanced screening and treatment on hepatitis C in the United States. Clin Infect Dis.

[3] Hope VD, Eramova I, Capurro D, Donoghoe MC (2014). Prevalence and estimation of hepatitis B and C infections in the WHO European region: a review of data focusing on the countries outside the European Union and the European free trade association. Epidemiol Infect.

[4] Bailey H, Turkova A, Thorne C (2017). Syphilis, hepatitis C and HIV in Eastern Europe. Curr Opin Infect Dis.

[5] Mukomolov S, Trifonova G, Levakova I, Bolsun D, Krivanogova E (2016). Hepatitis C in the Russian Federation: challenges and future directions. Hepat Med.

[6] Thrift AP, El-Serag HB, Kanwal F (2017). Global epidemiology and burden of HCV infection and HCV-related disease. Nat Rev Gastroenterol Hepatol.

[7] Justice ARFfHaS. Hepatitis C in Russia: an epidemic of negligence. http://en.rylkov-fond.org/blog/hcv/hcvrus/ 2014.

[8] Abdourakhmanov DT, Hasaev AS, Castro FJ, Guardia J. Epidemiological and clinical aspects of hepatitis C virus infection in the Russian Republic of Daghestan. Eur J Epidemiol. 1998;14:549–53. 10.1023/a:1007486002349 9794121

[9] Reshetnikov OV, Khryanin AA, Teinina TR, Krivenchuk NA, Zimina IY (2001). Hepatitis B and C seroprevalence in Novosibirsk, western Siberia. Sex Transm Infect.

[10] Dobrodeeva LK, Kornienko EB, Petrenya NN, Lutfalieva GT, Schegoleva LS, Demeneva LV, et al. A unique seroepidemiological pattern of HBV, HCV and HTLV-I in Nenets and Komi in northwestern Russia. Asian Pac J Cancer Prev. 2005;6:342–45 16235997

[11] Heimer R, Eritsyan K, Barbour R, Levina OS (2014). Hepatitis C virus seroprevalence among people who inject drugs and factors associated with infection in eight Russian cities. BMC Infect Dis.

[12] Armstrong GL, Wasley A, Simard EP, McQuillan GM, Kuhnert WL (2006). The prevalence of hepatitis C virus infection in the United States, 1999 through 2002. Ann Intern Med.

[13] Smith BD, Morgan RL, Beckett GA, Falck-Ytter Y, Holtzman D, Teo CG (2012). Centers for Disease Control and Prevention (CDC). Recommendations for the identification of chronic hepatitis C virus infection among persons born during 1945-1965. MMWR Recomm Rep.

[14] WHO (2017). Guidelines on hepatitis B and C testing.

[15] Gerlach JT, Diepolder HM, Zachoval R, Gruener NH, Jung MC, Ulsenheimer A (2003). Acute hepatitis C: high rate of both spontaneous and treatment-induced viral clearance. Gastroenterology.

[16] Galli C, Julicher P, Plebani M (2018). HCV core antigen comes of age: a new opportunity for the diagnosis of hepatitis C virus infection. Clin Chem Lab Med.

[17] WHO. Global health sector strategy on viral hepatitis 2016-2021. Geneva: World Health Organization; 2016.

[18] Consolidated strategic information guidelines for viral hepatitis: planning and tracking progress towards elimination. Geneva: World Health Organization; 2018. Licence: CC BY-NC-SA 3.0 IGO.

[19] Hajian-Tilaki K (2011). Sample size estimation in epidemiologic studies. Caspian J Intern Med.

[20] Kichatova VS, Kyuregyan KK, Soboleva NV, Karlsen AA, Isaeva OV, Isaguliants MG, Mikhailov MI (2018). Frequency of interferon-resistance conferring substitutions in amino acid positions 70 and 91 of Core protein of the Russian HCV 1b isolates analyzed in the T-cell Epitopic context. J Immunol Res.

[21] The Polaris Observatory HCV Collaborators (2017). Global prevalence and genotype distribution of hepatitis C virus infection in 2015: a modelling study. Lancet Gastroenterol Hepatol.

[22] Mariano A, Scalia Tomba G, Tosti ME (2009). Estimating the incidence, prevalence and clinical burden of hepatitis C over time in Italy. Scand J Infect Dis.

[23] Davis GL, Alter MJ, El-Serag H (2010). Aging of the hepatitis C virus (HCV)-infected persons in the United States: a multiple cohort model of HCV prevalence and disease progression. Gastroenterology.

[24] Falade-Nwulia O, Irvin R, McAdams-Mahmoud A (2016). Senior center-based hepatitis C screening in Baltimore. Open Forum Infect Dis.

[25] Kyuregyan КК, Soboleva NV, Karlsen АА (2019). Dynamic changes in the prevalence of hepatitis C virus in the general population in the republic of Sakha (Yakutia) over the last 10 years. Infect Dis.

[26] European Association for the Study of the Liver (2017). EASL recommendations on treatment of hepatitis C 2016. J Hepatol.

[27] Kuo YH, Chang KC, Wang JH, Tsai PS, Hung SF, Hung CH (2012). Is hepatitis C virus core antigen an adequate marker for community screening?. J Clin Microbiol.

[28] Reyes-Méndez MÁ, Juárez-Figueroa L, Iracheta-Hernández P, Medina-Islas Y, Ruiz-González V (2014). Comparison of two diagnostic algorithms for the identification of patients with HCV viremia using a new HCV antigen test. Ann Hepatol.

[29] Furlini G, Gelsomino F, Galli S, Favero S, Galli C (2017). Prevalence of anti-HCV and active HCV infection in an Italian hospital population. Clin Chem Lab Med.

[30] Gramenzi A, Conti F, Cammà C (2012). AISF Hepa elder study group. Hepatitis C in the elderly: a multicentre cross-sectional study by the Italian Association for the Study of the liver. Dig Liver Dis.

